# Prevalence and outcome of contrast-induced nephropathy in major trauma patients

**DOI:** 10.1007/s00068-020-01496-w

**Published:** 2020-09-19

**Authors:** Julian Alexander Kelemen, Alexander Kaserer, Kai Oliver Jensen, Philipp Stein, Burkhardt Seifert, Hans-Peter Simmen, Donat R. Spahn, Hans-Christoph Pape, Valentin Neuhaus

**Affiliations:** 1 Division of Trauma Surgery, Department of Surgery, University Hospital Zurich, University of Zurich, Raemistrasse. 100, 8091 Zurich, Switzerland; 2 Institute of Anesthesiology, University and University Hospital Zurich, Raemistrasse 100, 8091 Zurich, Switzerland; 3 Institute of Epidemiology, Biostatistics and Prevention, University of Zurich, Zurich, Switzerland; 4 Institute of Anesthesiology, Cantonal Hospital Winterthur, Brauerstrasse 15, 8400 Winterthur, Switzerland; 5 Department of Biostatistics at Epidemiology, Biostatistics and Prevention Institute, University of Zurich, Hirschengraben 84, 8001 Zurich, Switzerland

**Keywords:** Computed tomography, Contrast, Nephropathy, Trauma

## Abstract

**Background:**

Contrast-induced nephropathy (CIN) has been well investigated in patients undergoing coronary angiography, but not in trauma patients. The main aim of this study was to determine the prevalence and to investigate independent risk factors for the development of CIN.

**Methods:**

Between 2008 and 2014, all pre-hospital intubated major trauma patients with documented serum creatinine levels (SCr) undergoing a contrast-enhanced whole-body CT at admission were retrospectively analyzed. CIN was defined as a relative increase in SCr > 25% over the baseline value or an absolute SCr increase of > 44 µmol/l within 72 h. Univariate and multivariable regression analyses were performed to identify significant risk factors. A *p* value of < 0.01 was considered statistically significant and a *p* value of 0.01–0.049 suggested evidence.

**Results:**

Of 284 analyzed patients, 41 (14%) met the criteria for CIN. There is suggestive evidence that age and lactate level influenced the development of CIN. Six patients (15%) had hemodialysis in the CIN-group and eight (3.3%) in the group without CIN. Complication and mortality rate was higher in patients with CIN (71% vs. 56% and 32% vs. 23%, respectively). CIN was not an independent risk factor for complications or mortality while controlling for age, gender, injury severity score, and lactate level. The length of stay was not affected by CIN.

**Conclusion:**

CIN occurs frequently in trauma patients, but is not an independent risk factor for complications or mortality. Therefore, contrast enhanced whole-body CT can safely be performed in trauma patients.

## Background

Contrast-induced nephropathy (CIN) is characterized as an acute renal injury after the administration of intravascular iodinated radio-contrast medium in the absence of any other etiology [1]. Although the histological morphology of CIN is well characterized, the pathophysiologic mechanism of renal injury is still not clear. Most likely a combination of reduced renal perfusion, a reduction in tubular flow and/or a direct tubular toxicity of contrast agent could lead to a decrease in glomerular filtration rate [1, 2]. CIN is commonly diagnosed by a 25% rise from baseline creatinine, or an absolute increase in creatinine of ≥ 44 µmol/l 24–48 h after administration of contrast [3–6].

The incidence of CIN varies from 0.6 to 2.3% in patients without any pre-existing renal impairment and rises up to 30% in the presence of individual risk factors [7]. Advanced age, diabetes mellitus, pre-existing renal impairment, periprocedural intravascular volume depletion, congestive heart failure or concomitant use of other nephrotoxic drugs have been shown to increase the risk to develop CIN [2, 3, 6, 8, 9]. CIN is the third leading cause of hospital acquired acute renal failure and associated with a significant increase in mortality [2, 3]. Hence, several strategies (e.g., pre- and post-hydration, administration of *N*-acetylcysteine) were proven to be more or less effective in the prevention of CIN [10], but are not feasible in every situation—especially in the treatment of severely injured patients. Further, the cause of creatinine increase in severely injured patients can be multifactorial (e.g., contrast-induced, hemorrhagic shock, blood transfusions, advanced age).

Whole-body computed tomography (CT) was shown to reduce mortality in polytraumatized patients, if integrated into early trauma care [11, 12]. Therefore, contrast enhanced whole-body CT is more and more routinely performed for the initial evaluation of severely injured patients. Although crucial in the care of traumatized patients, only a few studies focus on the incidence, the impact and the clinical implications of CIN in those patients and the risk factors are discussed controversially [13–15]. Finigan et al. could not identify any risk factor for CIN in their work [14] and Kim et al. reported age ≥ 65 years and ISS ≥ 25 to be independently associated with acute renal injury, but not intravenous administered contrast medium [15]. The STARSurg Collaborative could not find any association between preoperative intravenous contrast administration and postoperative acute kidney injury after major gastrointestinal surgery [16].

To improve the understanding of CIN and its impact on severely injured patients, we conducted a retrospective analysis in our Level 1 trauma center. The main aim of this study was to determine prevalence of and to identify independent risk factors for CIN. Additionally, we assessed the clinical impact of CIN on the need of hemodialysis, complications rates, length of stay and mortality.

## Materials and methods

This retrospective cohort study was approved by the local ethics committee (Kantonale Ethikkommission Zürich, Switzerland, KEK ZH No. 2011-0382) and adheres to the Strengthening the Reporting of Observational Studies in Epidemiology (STROBE) recommendations for cohort studies [17, 18].

### Study design and participants

We included all adult, pre-hospital intubated trauma patients admitted to the emergency department of the University Hospital Zurich between October 2008 and December 2014 who received a contrast enhanced whole-body CT directly on arrival (*n* = 350). Patients with missing initial or follow-up (within 72 h) serum creatinine levels (sCr) were excluded from the study (*n* = 66). The whole-body CT algorithm of the University Hospital Zurich was changed at the end of 2014. To avoid a potential study bias, we limited our analysis to the above-mentioned period. A total of 284 patients met inclusion criteria and were further analyzed.

### Setting

Severely injured patients are admitted to the resuscitation area of our level I trauma center. In the division of trauma surgery, a standardized clinical approach is provided in the resuscitation area. The standard diagnostic algorithm includes a native head and neck CT followed by a primary contrast enhanced (100 ml iso-osmolar, non-ionic iodinated contrast material [300 mg iodine per millilitre, iopromide (Ultravist^®^; Bayer Healthcare, Leverkusen, Germany)] followed by a saline flush of 30 ml [19]) chest and abdomen CT during the whole study period. After initial stabilization in collaboration with the anesthesiologists, damage control or early total care surgery is performed [20], and patients are subsequently transferred to the intensive care unit for further treatment.

### Variables and data collection

Demographics (age, gender comorbidities), injury severity score (ISS), complications, length of stay (LOS), need for hemodialysis, and death were extracted from the electronic medical record (KISIM, Cistec AG, Zürich, Switzerland). Extracted laboratory values included hemoglobin and fibrinogen level at admission (g/l), initial base excess (mmol/l) as well as serum lactate (mmol/l), initial and follow-up SCr levels. In addition, the following data were extracted from the anesthesia records until the admission to the ICU: volume of resuscitation fluids like crystalloid solution (in liters), the use of allogeneic blood products such as packed red blood cells (RBC, in numbers), fresh frozen plasma (FFP, yes/no), platelet concentrates (yes/no) and the use of fibrinogen (yes/no).

Contrast-induced nephropathy (CIN) was defined as a relative increase in serum creatinine > 25% of the baseline value or an absolute increase > 44 µmol/l within 72 h [6].

Complications were defined as the presence of acute delirium, myocardial infarction, deep vein thrombosis, pulmonary embolism, urinary tract infection, surgical site infection or complications such as pneumonia, sepsis, SIRS, cerebrovascular incidents or coagulopathy during the hospitalization [21].

### Study endpoints

The main goal of this study was to determine prevalence of and to identify independent risk factors for CIN.

Additionally, we assessed the clinical impact of CIN on the need of hemodialysis, complications rates, length of stay and mortality.

### Statistical analyses

Categorical data are reported as frequency and percent and numerical data as mean ± standard deviation (SD). The Chi‐square and Fisher’s exact test were used to compare categorical data and the Mann–Whitney *U*-test for numerical data. Age, gender, Injury Severity Score for anatomic severity, and lactate level for physiologic severity were entered in multiple regression analysis as suggested by Haider et al. (“Bare minimum”) [22]. In addition, CIN was forced into the models where applicable. Model fit was assessed using the Hosmer–Lemeshow test. The Glasgow Coma Scale and need for ventilator use were not used in these models since all patients were intubated at arrival. Lactate level was skewed to the right and therefore logarithmically transformed. All statistical analyses were performed by IBM SPSS Statistics 23 (IBM Corp., Armonk, NY, USA). Missing values were not entered into the analysis. A *p* value of < 0.01 was considered statistically significant, a *p* value of 0.01–0.049 showed suggestive evidence [23].

## Results

### Prevalence of and independent risk factors for CIN

Prevalence of CIN was present in 41 out of 284 patients (14%) (Table 1). Patients suffering from CIN had a significant lower hemoglobin level at admission (*p* = 0.002), were on average 9 years older (*p* = 0.019), had a slightly lower creatinine (*p* = 0.026) and lactate level (*p* = 0.036) at admission. Systolic blood pressure at admission was similar in both groups (*p* = 0.094). Patients with CIN received slightly more crystalloids (*p* = 0.042), fibrinogen (*p* = 0.027), and red blood cells (*p* = 0.017). In multivariate analysis, there is suggestive evidence that age (*p* = 0.035) and lactate level (*p* = 0.034) influenced the development of CIN (*R*^2^ = 0.071) (Table 2).

**Table 1:** Summary of patient characteristics between the CIN and no CIN group

	No CIN	CIN	*p* value
	*n* = 243 (86%)	*n* = 41 (14%)	
Age (years)	46 ± 22	55 ± 23	0.019
Gender (male)	192 (79%)	29 (71%)	0.238
Comorbidities	145 (60%)	25 (61%)	0.875
Blunt trauma	235 (97%)	40 (98%)	0.673
Injury Severity Score	28 ± 17	30 ± 16	0.296
AIS head	3.1 ± 1.8	3.2 ± 1.8	0.733
AIS face	0.7 ± 1.1	0.6 ± 1.2	0.765
AIS chest	1.5 ± 1.8	1.6 ± 1.7	0.771
AIS abdomen	0.6 ± 1.2	0.7 ± 1.3	0.719
AIS extremity	1.2 ± 1.4	1.5 ± 1.6	0.316
AIS integument	0.9 ± 0.9	0.9 ± 0.8	0.961
Heart rate at admission (bpm)	88 ± 24	86 ± 22	0.513
Systolic blood pressure at admission (mmHg)	121 ± 28	112 ± 21	0.094
Serum creatinine (µmol/l)	81 ± 29	73 ± 26	0.026
Hemoglobin (g/l)	11.7 ± 2.4	10.4 ± 2.7	0.002
Fibrinogen (g/l)	2.1 ± 0.7	2.2 ± 1.1	0.645
Lactate level (mmol/l)	2.7 ± 2.6	2.0 ± 1.7	0.036
Base excess (mmol/l)	− 4.2 ± 4.6	− 4.2 ± 4.5	0.800
Crystalloid (l)	2.2 ± 2.9	2.6 ± 2.4	0.042
Red blood cells (*n*)	1.3 ± 4.9	2.3 ± 4.7	0.017
Fresh frozen plasma	13 (5.4%)	4 (9.8%)	0.287
Platelets	18 (7.5%)	7(17%)	0.069
Fibrinogen	80 (33%)	21 (51%)	0.027
Hemodialysis	8 (3.3%)	6 (15%)	0.008
Complications	136 (56%)	29 (71%)	0.076
Length of stay (days)	14 ± 13	18 ± 20	0.407
Mortality	55 (23%)	13 (32%)	0.208

*CIN* contrast-induced nephropathy

**Table 2:** Risk factors for CIN

	OR	95% CI		*p* value
Age (years)	1.017	1.001	1.033	0.035
Injury Severity Score	1.008	0.988	1.029	0.430
Gender (Male)	0.806	0.370	1.760	0.589
Lactate level (logarithmic)	0.269	0.080	0.906	0.034

Multivariable logistic regression analysis with following explanatory variables: age, gender, Injury Severity Score for anatomic severity, and logarithmically transformed lactate level for physiologic severity

*CI* confidence interval, *OR* odds ratio

### Hemodialysis

Hemodialysis was necessary in 6 out of 41 CIN patients (15%) and 8 out of 243 patients (3.3%) without CIN (*p* = 0.008) (Table 1). The use of fresh frozen plasma (*p* = 0.006) and platelets (*p* = 0.004) were significantly, Injury Severity Score (*p* = 0.034), amount of infused crystalloids (*p* = 0.012), numbers of RBC (*p* = 0.040) and use of fibrinogen (*p* = 0.023) were slightly associated with hemodialysis. The low numbers of hemodialysis did not allow multivariate analysis.

### Complications

Complications during hospital stay occurred in 29 CIN patients (71%) and in 136 patients (56%) without CIN (*p* = 0.076). Pneumonia (10/29), wound infections (4/29), deep vein thrombosis (4/29) and sepsis (3/29) were the most common complications in patients suffering from CIN. Injury Severity Score (*p* < 0.001), hemoglobin (*p* = 0.001) and fibrinogen at admission (*p* = 0.002), base excess (*p* = 0.001), amount of crystalloids (*p* < 0.001), numbers of RBC (*p* = 0.001), use of fibrinogen (*p* < 0.001) and FFP (*p* = 0.008) were significantly associated with the development of complications. However, preexisting comorbidities (*p* = 0.024) were only slightly associated with the development of complications. In multivariate analysis, ISS was a risk factor for complications (*p* = 0.004), but not CIN (*R*^2^ = 0.083) (Table 3).

**Table 3:** Risk factors for any complications

	OR	95% CI		*p* value
CIN	2.029	0.960	4.291	0.064
Lactate level (logarithmic)	2.027	0.867	4.737	0.103
Injury Severity Score	1.025	1.008	1.042	**0.004**
Age (years)	0.998	0.986	1.010	0.730
Gender (male)	0.939	0.511	1.727	0.840

Bold values indicate significant values

Multivariable logistic regression analysis with following explanatory variables: age, gender, Injury Severity Score for anatomic severity, and logarithmically transformed lactate level for physiologic severity

*CI* confidence interval, *CIN* contrast-induced nephropathy, *OR* odds ratio

### Length of hospital stay

The length of hospital stay was not affected by CIN (18 ± 20 with vs. 14 ± 13 days without CIN, *p* = 0.407) (Table 1).

### Mortality

In-hospital mortality was 32% in patients with and 23% in patients without CIN (*p* = 0.208) (Table 1). Age (*p* < 0.001), comorbidities (*p* < 0.001), ISS (*p* < 0.001), hemoglobin at admission (*p* < 0.001), base excess (*p* < 0.001), amount of RBC (*p* = 0.006), use of FFP (*p* = 0.007) or platelets (*p* < 0.001) were significantly, lactate level (*p* = 0.044) slightly associated with death. CIN was not an independent risk factor for mortality (*R*^2^ = 0.435). However, age (*p* < 0.001) and a higher ISS (*p* < 0.001) were independent predictors for mortality (Table 4).

**Table 4:** Risk factors for mortality

	OR	95% CI		*p* value
CIN	1.192	0.480	2.962	0.706
Injury Severity Score	1.098	1.066	1.130	**<** **0.001**
Lactate level (logarithmic)	1.618	0.533	4.916	0.396
Gender (male)	1.051	0.446	2.480	0.909
Age (years)	1.030	1.013	1.047	**<** **0.001**

Bold values indicate significant values

Multivariable logistic regression analysis with following explanatory variables: age, gender, Injury Severity Score for anatomic severity, and logarithmically transformed lactate level for physiologic severity

*CI* confidence interval, *CIN* contrast-induced nephropathy, *OR* odds ratio

## Discussion

Most of the research about CIN has been performed in patients undergoing percutaneous coronary interventions (PCI). To our knowledge, this study is one of few studies investigating CIN in the population of severe trauma and the only one in an European trauma center [24]. We thus intentionally focused on severely injured and already intubated patients. Due to our standardized diagnostic approach, they are most likely to undergo immediate contrast enhanced whole-body CT regardless of any pre-existing medical condition (e.g., renal insufficiency, diabetes mellitus), allergies or specific injuries. The prevalence of CIN was 14% in this population and we could identify lactate level as well as age as independent risk factors. Patients suffering from CIN were more likely to need hemodialysis (4.7 times), but the complication and mortality rate as well as length of hospital stay were not significantly affected by CIN.

The prevalence of CIN was 14% in our study population, which is slightly higher than described by other authors. Comparable studies found a prevalence ranging from 2.1 to 5.1% [3, 4, 13, 25]. Other studies in non-trauma patients reported a prevalence of 0.8–4.7% [26, 27]. The reason for our higher prevalence is probably due to our cohort compromising only severely injured patients. Age was a suggestive risk factor for CIN in our cohort. This is comprehensible and in accordance with other studies [28]. A lower lactate level was also a suggestive risk factor. This seems counterintuitive. However, the authors interpret this due to the increased need of volume and transfusions of these patients, as allogenic blood products are associated with a number of harmful side effects [29, 30]. The question remains if an aggressive volume and transfusion management really increases the risk of CIN. Low hemoglobin level on admission was significantly associated with nephropathy, as also described by Xu and colleagues in patients undergoing coronary angioplasty or PCI [31]. Other authors did not find anemia to be a risk factor for CIN, at least in cardiac patients [32, 33]. Banda et al. found that anemia was not a risk factor for CIN, but CIN in combination with low hemoglobin levels doubled the mortality in their population [34]. Likewise, there is suggestive evidence that the amount of transfused red blood cell units is associated with CIN. There is possibly an exponentiating effect. Red blood cell transfusion of itself is a clear risk factor for acute kidney injury; in a meta-analysis by Karkouti et al., every unit of red blood cells increased the risk for acute kidney injury by 10–20% in cardiac surgery patients [35].

The development of nephropathy in polytraumatized patients is an intricate process. Vassiliu and his colleges analyzed trauma patients after angiographic embolization and found that age, hypovolemia, renal trauma and severe injuries were no risk factors for CIN in their population [36]. In contrast, Matsushima et al. found a higher ISS to be an independent risk factor for developing CIN [37]. The literature shows several strategies to prevent CIN. Because of the feasibility in this setting and the lack of doubtless proof [10] no routine administration of n-acetylcysteine was performed in our hospital.

The hemodialysis rate was 15%. Other studies found 0.3–7% of patients requiring hemodialysis after the administration of contrast agents [4, 38]. The presence of CIN significantly increased the risk for hemodialysis in our population. One study found a higher rate of hemodialysis in patients developing CIN but was not able to identify CIN as a significant risk factor [39]. In our cohort, a higher ISS, an increased amount of crystalloids, allogeneic blood transfusion and procoagulant agents were associated with an increased need for hemodialysis. As with the nephropathy, the question remains about the causality.

Severely injured patients had a high complication rate, independent of CIN. Most of these complications were infections (pneumonia, cystitis) or wound complications (seroma, surgical site infection). The ISS was the most important risk factor for complications, which is consistent with the results of many other trauma studies. Patients suffering from CIN had not a higher risk of experiencing complication during their hospitalization.

Age and ISS influenced the mortality rate in our study, but not CIN. Other studies found an increased 1-year mortality in patients suffering from CIN after cardiac interventions. But it remains unclear if this due to CIN or the present underlying conditions and comorbidities, asking for the contrast-enhanced CT [40, 41].

There are some limitations to this study. Due to the retrospective design of our study we can only deduct association and not causation. There may be some confounders which we cannot detect and correct retrospectively. The whole-body CT algorithm of the University Hospital Zurich was changed at the end of 2014. To avoid a potential study bias, we limited our analysis to the period from 2008 to the end of 2014. We intentionally focused on prehospital intubated patients due to our pre-defined, consistent diagnostic approach. All intubated trauma patients underwent immediately contrast-enhanced whole-body CT regardless of the injury pattern; the presence of a known renal impairment, allergies, or SCr levels was never known prior to the CT scan in these patients. Not intubated trauma patients may have undergone a different diagnostic approach due to the possibility to take a medical history at admission and to consider contraindication to perform a contrast enhanced whole-body CT scan. Available long-term data were incomplete and did not allow us to perform follow-up examinations to detect persistent renal failure or injury. Last, our analyzed patient cohort was not big enough to control for more than five factors in multivariate analysis.

We conclude that even in severely injured patients it is safe to perform contrast enhanced whole-body CT without further harming these patients. Even if CIN occurs, a benign course is likely and the patient’s outcome, especially mortality and length of hospital stay are not affected. Further research is needed to investigate the effect of fluid and blood management on the development of CIN and the long-term outcome of patients affected by CIN.

## Abbreviations

AKI: Acute kidney injury
CIN: Contrast-induced nephropathy
CPR: Cardiopulmonary resuscitation
FFP: Fresh frozen plasma
HES: Hydroxyl ethyl starches
ISS: Injury severity score
PCI: Percutaneous coronary intervention
RBC: Packed red blood cells
SCr: Serum creatinine levels
